# A Cure by Imagination

**Published:** 1881-10

**Authors:** 


### A CURE BY IMAGINATION.

At a large hotel the not uncommon dilemma arose of there being only one room in the house vacant when two visitors required accommodations for the night. It was a double-bedded chamber, or was soon converted into such, and the two guests, who were both commercial travelers, agreed to share it. One of these gentlemen was a confirmed hypochondriac, and greatly alarmed his companion by waking him up in the middle of the night, gasping for breath. "Asthma," he panted out, "I am subject to these spasmodic attacks. Open the window quickly; give me air!" Terrified beyond measure, the other jumped out of bed. But the room was pitch dark; he had no matches, and he had forgotten the position of the window. "For heaven's sake, be quick!" gasped the invalid. "Give me more air, or I shall choke!" At length, by dint of groping wildly and upsetting half the furniture in the apartment, the window was found; but it was an old-fashioned casement, and no hasp or catch was to be discovered.

"Quick, quick; air, air!" implored the apparently dying man. "Open it, break it, or I shall be suffocated!" Thus adjured, his friend lost no more time, but seizing a boot, smashed every pane, and the sufferer immediately experienced great relief. "Oh, thank you, a thousand thanks. Ha!" he exclaimed, drawing deep sighs, which testified to the great comfort he derived, "I think in another moment I should have been dead!" And when he had sufficiently recovered, and had expressed his heartfelt gratitude, he described the intense distress of these attacks and the length of time he had suffered from them. After awhile both fell asleep again, devoutly thankful for the result. It was a warm summer night, and they felt no inconvenience from the broken window, but when daylight relieved the pitch darkness of the night the window was found to be still entire! Had invisible glaziers been at work already, or was the episode of the past night only a dream? No; for the floor was still strewn with the broken glass. Then, as they looked round the room in amazement, the solution of the mystery presented itself in the shape of an antiquated bookcase, whose latticed glass doors were a shattered wreck. The spasmodically attacked one was cured from that moment. So much for imagination.—*Chambers' Journal.*

---

A MODERATE gale travels at the rate of sixteen feet in a second.

